# Periodized Resistance Training for Enhancing Skeletal Muscle Hypertrophy and Strength: A Mini-Review

**DOI:** 10.3389/fphys.2019.00013

**Published:** 2019-01-23

**Authors:** Jonathan W. Evans

**Affiliations:** University of Ottawa, Ottawa, ON, Canada

**Keywords:** periodization, resistance training, muscle hypertrophy, strength, exercise

## Abstract

Prescribing the proper resistance training (RT) program is critical to optimize skeletal muscle hypertrophy and strength. Periodization is a strategy that entails planned manipulations of training variables to maximize fitness adaptations while minimizing the risk of overtraining. Multiple meta-analyses have shown periodized RT to be superior to non-periodized RT for enhancing muscular strength. These findings are consistent irrespective of training status or training volume. Both the linear model and the undulating model are effective for enhancing strength, although a greater benefit might be achieved through the undulating model. Despite the suggested superiority of periodized RT for strength development, some authors suggest that this might be a consequence of the study designs employed rather than the nature of periodized training. In addition, several limitations exist in the periodization literature, making it difficult to accurately assess the efficacy of periodized RT. With regard to enhancing skeletal muscle hypertrophy, both the undulating model and the linear model appear equally effective; however, this conclusion can only be generalized to untrained populations. When comparing periodized RT to non-periodized RT programs, the research is unclear on whether periodized RT is necessary to maximize skeletal muscle hypertrophy.

## Introduction

Muscular strength can be defined as a muscle’s ability to exert force on an external resistance (Suchomel et al., 2018). While muscular strength is suggested to be a critical attribute for many athletic disciplines (Suchomel et al., 2016), it is also an essential component of functionality in daily living (Kraemer et al., 2002; Hunter et al., 2004; Westcott, 2012). Young, old, and athletic individuals can utilize resistance training (RT) as a means to improve strength (Peterson et al., 2004; Faigenbaum et al., 2009; Borde et al., 2015), and the magnitude of strength improvement is influenced by the structure of the training program (American College of Sports Medicine, 2009). In addition to increasing muscular strength, RT is used by many individuals as a means to enhance skeletal muscle hypertrophy (i.e., enlargement of skeletal muscle tissue). For instance, success in sports such as bodybuilding is dependent on obtaining maximal levels of muscle mass while carrying minimal fat mass (Helms et al., 2015). Considering the importance that strength and muscle mass hold in regard to athletic success and overall well-being, implementation of the proper RT program is critical to optimize these attributes. Periodization appears to be an effective means for enhancing strength (Rhea and Alderman, 2004; Williams et al., 2017); however, its influence on muscle hypertrophy is less clear. The purpose of this mini-review is to examine the current evidence regarding the application of periodized RT for enhancing muscular strength and skeletal muscle hypertrophy. Limitations and suggestions for future research will be addressed, as this will better enable researchers to accurately assess the efficacy of periodized RT.

## An Overview of Periodization

### Defining Periodization

Periodization can be defined as the planned manipulation of training variables to optimize performance at appropriate time points, manage fatigue, and prevent stagnation (Plisk and Stone, 2003). These variables (such as volume, intensity, and exercise selection) are varied in a cyclical fashion across training cycles to promote peak fitness levels for targeted competitions (Haff, 2004). The training cycles that are incorporated into the periodized plan consist of the macrocycle, which usually lasts a year, the mesocycle, which may last a month, and the microcycle, which may last a week (Turner, 2011). As noted by Turner (2011), substantial variability exists between the lengths of each training cycle, which will be dependent on the athlete’s goals and competition schedule. In addition, taper periods are commonly incorporated into the training plan to allow the athlete to “peak” in readiness for a competition (Haff, 2004). For strength athletes, these phases may consist of reductions in training volume with maintained or slight increases in intensity (Pritchard et al., 2015). Unloading weeks (which are also characterized by reductions in training volume) may be implemented at the end of each mesocycle to promote further recovery and adaptation from training (Turner, 2011).

### A Physiological Basis for Periodized Training

For a training program to remain effective, it must continually overload the neuromuscular system (Kraemer and Ratamess, 2004). It has been suggested that variations in training stimuli are necessary to optimize strength adaptations (Kraemer et al., 1988; Tan, 1999), since variation may force the neuromuscular system to continually adapt to unaccustomed stress (Rhea and Alderman, 2004). Conversely, lengthy time periods of loading that are devoid of variation may result in fatigue and stagnation (Williams et al., 2018). Therefore, one of the purposes of periodization is to implement structured variability into training to offset the negative outcomes that may occur through the stress of linear loading. Support for this notion is demonstrated in a recent study which compared the effects of two periodized training regimens to a non-periodized regimen over 12 weeks of RT (De Souza et al., 2018). While strength gains favored the non-periodized group after the initial 6 weeks of training, further strength gains were minimal (1.5%) in this group during weeks 6–12. Conversely, both periodized groups were able to enhance strength during this time span (9.4 and 6.9%). These findings indicate that the effectiveness of a non-periodized training program may only last a few months before stagnation occurs.

In addition to incorporating variability within the overall training structure, many periodized training plans implement taper phases prior to important competitions (Smith, 2003; Turner, 2011). These phases consist of reduced training loads with the intent of restoring the athlete from training-induced emotional and physiological stress (Mujika and Padilla, 2000). The basis for tapering relies heavily on the fitness-fatigue model, which suggests that physical training will produce a simultaneous build-up of fitness and fatigue after-effects (Chiu and Barnes, 2003). Importantly, it is the difference between these after-effects that may determine athletic preparedness (Chiu and Barnes, 2003). DeWeese et al. (2015) suggest that when training volume is reduced, fatigue will dissipate at a faster rate than fitness, which may necessitate the need for a taper phase prior to a competition. While current research supports the efficacy of tapering for maximal strength (Pritchard et al., 2015), the mechanisms responsible for this effect are unclear. Some authors suggest that tapering may reduce muscle damage and neuromuscular fatigue, thereby promoting muscular strength improvements (Murach and Bagley, 2015). Importantly, however, available data on this topic are limited; more research is required to elucidate potential mechanisms behind the effect of tapering on strength enhancement.

### Periodization Models

While the tenets of periodization remain constant, there are several ways that a periodized training plan can be implemented. The most common periodization model is the traditional (or “linear”) periodization model (LP). This model initiates with high training volumes and low intensities and gradually progresses toward low training volumes and high intensities over the course of several months (Fleck, 2011). It should be noted that the term “linear periodization” is a misnomer, considering the training plan does not end at the completion of the high-intensity phase. All periodization models are cyclical in nature; therefore, it is inaccurate to depict any periodization model as “linear” (Stone and Wathen, 2001). In addition to the “linear” model, the reverse-linear periodization model has been proposed, which is nearly identical to the linear model except that it is run backward (Helms et al., 2015). This model initiates with low training volumes and high intensities and gradually progresses toward high training volumes and low intensities. One of the more heavily-researched periodization models is the undulating (or “non-linear”) periodization model (UP). This model entails more frequent variations in loading than the previous two models. Specifically, loading zones may vary on a daily, weekly, or bi-weekly basis (Buford et al., 2007). It should be noted, however, that UP and LP are not mutually exclusive. For instance, UP models often incorporate linearity to coincide with upcoming competitions. Specifically, while UP entails frequent variations in loading, repetition schemes can progress from high (hypertrophy-oriented) to low (strength/power-oriented) over the course of several training phases (Poliquin, 1988).

## Periodization for Enhancing Muscular Strength

### Support for the Efficacy of Periodized Resistance Training

Since its introduction into strength and conditioning literature, periodization has received much attention for its beneficial effects on strength performance. Support for the efficacy of periodized RT has been demonstrated in two meta-analyses, both of which showed periodized RT to be superior to non-periodized RT for enhancing maximal strength (Rhea and Alderman, 2004; Williams et al., 2017). Importantly, both analyses found the superiority of periodized RT to be consistent irrespective of training volume or training status. The authors of each analysis note that the superior effect of periodized RT might be a conservative estimate, since the studies included in each analysis were short in duration. In the more recent meta-analysis by Williams et al. (2017), the majority of studies lasted less than 16 weeks. Since periodization is designed to be a long-term approach to training (Pedemonte, 1986; Fry et al., 1991; Stone et al., 1999), it is possible that the superior effect of periodized RT may be even greater than presented in each analysis.

### The Effect of Periodization Model on Enhancing Muscular Strength

The notion that a specific periodization model might elicit superior strength improvements compared to other models has been subject to a great deal of research. Some authors have suggested that UP may produce superior strength gains since it incorporates more frequent variations in loading (Poliquin, 1988; Rhea et al., 2002). Since the LP model generally entails lengthy time periods spent in a specific loading zone, the lifter might quickly adapt to the training stimulus, which may result in stagnation. Conversely, since the UP model varies the training stimulus more frequently, the lifter may be forced to continually adapt to the unaccustomed stress. A meta-analysis by Harries et al. (2015) found no significant difference between UP and LP for eliciting maximal strength gains; however, the authors did show a trend favoring UP as being more beneficial for leg press strength (mean difference = 25.93 [95% confidence interval -2.48 to 54.35] kg, *Z* = 1.79 [*p* = 0.07]). A subsequent meta-analysis by Caldas et al. (2016) found UP to produce significantly greater improvements in maximal strength than LP. The different results may be due to the larger data pool included in the analysis by Caldas et al. (2016). In the meta-analysis by Williams et al. (2017), it was found that UP produced superior strength gains to LP. As Williams et al. (2017) stress, however, the primary objective of their analysis was to determine the effects of periodized versus non-periodized RT programs for strength development. Therefore, their analysis did not include studies which solely compared different periodization models against each other. Considering these findings, it is possible that UP might be ideal for optimizing maximal strength.

### Variation or Specificity?

Periodization theory suggests that training variation is essential to maximize fitness adaptations (Haff, 2004). In the meta-analysis by Williams et al. (2017), the authors conclude that, “variation in training stimuli appears to be vital for increasing maximal strength.” However, close inspection of the studies included in their analysis reveals that the periodized groups trained with higher intensities prior to testing compared to the non-periodized groups (Nunes et al., 2018). This has led some authors to suggest that the superior results experienced by the periodized groups may have been due to the principle of specificity rather than the varied nature of periodized training (Nunes et al., 2018). Indeed, research has demonstrated that training with heavy loads can elicit greater one-repetition maximum (1RM) improvements compared to training with light loads (Schoenfeld et al., 2017). These findings are in accordance with the principle of specificity, since training with near-maximal loads is highly specific to performing a 1RM test (Buckner et al., 2017). Considering this point, Nunes et al. (2018) suggest that to truly determine if the improvements seen through periodized RT are due to training variation (and not the principle of specificity), studies should designate high-intensity loading zones (1–5 repetition range) to the non-periodized groups, while the periodized groups train across a variety of loading zones.

A recent study attempted to address this issue by comparing the effects of “daily max” training to a LP protocol in competitive powerlifters (Androulakis-Korakakis et al., 2018). The daily max group completed single sets of one repetition with near-maximal loads (9–9.5 rating of perceived exertion) during each training session. Conversely, the LP group performed multiple sets with loads ranging from 70–93% 1RM. After 10 weeks of training, two out of the three subjects in the LP group increased their powerlifting total (2 and 6.5%), whereas one subject experienced no change. In the daily max group, two out of the five subjects increased their powerlifting total (4.8 and 4.2%) whereas three subjects experienced a decrease in their powerlifting total (-0.5, -3.4, and -5%). While the superior outcomes experienced in the LP group would appear to confirm the benefits of periodized training, it must be stressed that this group trained with substantially more volume than the daily max group. Considering the influence that training volume has on strength development (Rhea et al., 2003; Krieger, 2009; Ralston et al., 2017), it is unclear whether the superior outcomes seen in the LP group were primarily due to training variation or training volume. As mentioned previously, chronic periods of loading that are devoid of variation may lead to fatigue and stagnation (Williams et al., 2018), thereby hindering strength performance. In this regard, it is possible that chronically high volumes of heavy loading might induce a state of overtraining that may be deleterious to neuromuscular adaptation (Stone et al., 1991). Since periodization theory suggests that the proper manipulation of volume and intensity may mitigate overtraining potential (Stone et al., 1991), future studies should compare these two training conditions (i.e., high training volumes with heavy loads versus volume-matched periodized RT) to accurately establish the effect of training variation on strength enhancement. In addition to the difference in training volume between groups, the low number of subjects included in the data analysis (*n* = 8) by Androulakis-Korakakis et al. (2018) warrants caution when interpreting the findings of this study. Furthermore, post-testing occurred in a powerlifting meet rather than a controlled study environment. This creates the possibility that the subjects’ tactics and mental states might have influenced the results. Future studies should address these issues to accurately assess the influence of training specificity and training variation on strength improvements from periodized RT.

## Periodization for Enhancing Skeletal Muscle Hypertrophy

### Is Periodized Resistance Training Necessary to Maximize Skeletal Muscle Hypertrophy?

As previously mentioned, many individuals engage in RT as a means to enhance skeletal muscle hypertrophy as well as strength. Only recently has research paid close attention to this attribute with regard to periodized RT programs. A recent systematic review analyzed 12 studies comparing periodized RT to non-periodized RT protocols for hypertrophy outcomes (Grgic et al., 2018). The authors concluded that while both program types yield similar results, the limited number of studies analyzed (with just two including trained subjects) makes it difficult to draw definitive conclusions. In addition, the authors of the review suggest that direct measures of muscle hypertrophy, such as MRI or ultrasound, may be required to elucidate potential differences between groups. Unfortunately, only three studies included in the review used either of these measurement tools, with one out of the three studies (Schoenfeld et al., 2016) suggesting a slight benefit to periodized RT.

It could be argued that the lack of differences found between training conditions may be due to short study durations. A 9-month study by Kraemer et al. (2003) compared the effects of UP to non-periodized RT in two groups of female tennis players. After the duration of the study period, it was found that the UP group experienced greater absolute changes in fat-free mass than the non-periodized group (3.3 ± 1.7 kg versus 1.6 ± 2.4 kg, respectively). While this study suggests that a long study duration may be required for differences in muscle hypertrophy to manifest between groups, a 6-month study by Hunter et al. (2001) did not find a superior effect of periodized RT over a non-periodized program in untrained older adults. As mentioned by Schoenfeld et al. (2016), the conflicting results may be due to the differences in study populations (young female tennis players versus inactive older adults). More research is required to investigate the effects of long-term periodization programs on skeletal muscle hypertrophy.

### The Effect of Periodization Model on Enhancing Skeletal Muscle Hypertrophy

In 1988, Poliquin proposed that the undulating model may offer a superior hypertrophic effect compared to the linear model, since the linear model entails lengthy time spans spent in a particular loading zone (Poliquin, 1988). Specifically, since LP models generally allocate a month or longer of training time to be spent in a “strength-power” training phase, the lifter might lose the muscle gains he or she achieved in the hypertrophy phase of training. Conversely, since the UP model incorporates hypertrophy sessions on a weekly or bi-weekly basis, it could be argued that this frequent exposure to hypertrophy-focused training would be more conducive for muscle gains. To this author’s knowledge, two meta-analyses have been conducted to compare the effects of LP to UP for enhancing skeletal muscle hypertrophy (Caldas et al., 2016; Grgic et al., 2017). While neither analysis found an advantage to either model for hypertrophy outcomes, it should be noted that many of the studies included in each analysis utilized strength-oriented program designs with hypertrophy measured as a secondary outcome. It is possible that different results might have occurred had all of the included studies implemented hypertrophy-focused training designs (such as higher repetition schemes used prior to testing). In addition to this issue, just one out of the 13 studies included in the analysis by Grgic et al. (2017) used trained subjects. Similarly, in the analysis by Caldas et al. (2016), six studies were examined for hypertrophy outcomes, with only one study using trained subjects. Considering trained individuals display a different physiological response to RT than untrained individuals (Damas et al., 2015), the results of these analyses may not be applicable to lifters with training experience.

### Targeting Different Muscle Fibers

Grgic and Schoenfeld (2018) suggested that skeletal muscle might respond to RT in a fiber type-specific manner. Specifically, low loads that promote more time under tension may produce greater hypertrophy of the type I muscle fibers, whereas high loads may preferentially target the type II muscle fibers (Grgic and Schoenfeld, 2018). While the authors concluded that there is limited available evidence to support this claim, they did cite research from Netreba et al. (2013) and Vinogradova et al. (2013) as support for the notion of fiber type-specific adaptations to RT. Considering this possibility, incorporating a combination of loading zones in a periodized fashion may be required to maximize the growth of both fiber types (Ogborn and Schoenfeld, 2014). An 8-week study by Schoenfeld et al. (2016) sought to determine if training across a wide spectrum of loading zones would result in superior muscle adaptations compared to a non-periodized group. The periodized condition was constructed as a UP model that incorporated loading zones of a 2–4 repetition maximum (RM) on Day 1, an 8–12 RM on Day 2, and a 20–30 RM on Day 3. The non-periodized group adhered to an 8–12 RM throughout the duration of the study. At the end of the study period, no significant differences were found between groups for hypertrophy or strength outcomes; however, effect sizes favored the UP group for increases in muscle thickness. While this study suggests a potential hypertrophic benefit to training across a wide spectrum of repetition ranges, it is unclear if the muscle gains experienced in the UP group occurred in a fiber type-specific manner. More research is needed to determine the effects of various loading strategies on different muscle fibers.

## Limitations and Directions for Future Research

While the current evidence supports the use of periodized RT for optimizing muscular strength, several limitations exist in the periodization literature. As mentioned previously, the majority of studies comparing periodized training to non-periodized training are short in duration (Cissik et al., 2008; Afonso et al., 2017). Considering periodization originated as a long-term approach to training, it is important that researchers conduct longer trials to accurately establish the efficacy of periodized RT programs. Furthermore, an objective of periodization is to promote peak levels of performance at designated time points (Naclerio et al., 2013). This may be accomplished through careful manipulations of volume and intensity in the weeks prior to testing. Interestingly, a review by Afonso et al. (2017) found that predictions regarding the timing and magnitude of fitness adaptations are generally non-existent in periodization studies. Rather than focusing on this aspect of periodization, most studies only use training variation to represent a periodized approach to training (Kiely, 2012; Afonso et al., 2017). This is not a complete representation of periodization; future studies should address this issue by implementing taper periods and unloading cycles into the training plans at appropriate time points.

There is a tendency in strength and conditioning research to differentiate periodization models from one another (for instance, UP is often viewed separately from LP). However, as mentioned previously, UP can incorporate linearity within the overall training plan. When examining the effects of periodized RT on enhancing muscular strength, it might be useful for researchers to integrate aspects of different periodization models into individual training plans, as this approach may provide advantages over rigid periodization structures. In addition to assessing strength adaptations, subjective fatigue questionnaires should be assigned to each treatment group to determine whether periodization is effective at minimizing subjective fatigue while optimizing mental engagement to training. As to the paper by Nunes et al. (2018), future studies comparing periodized RT to non-periodized RT should assign higher training intensities (1–5 repetition range) to the non-periodized groups while the periodized groups train across a variety of intensities (on a volume-equated basis).

When examining the effects of periodized RT on skeletal muscle hypertrophy, there is a dearth of studies on trained subjects. Furthermore, many periodization studies that analyze hypertrophy outcomes tend to implement strength-oriented programs with hypertrophy measured as a secondary outcome. Constructing hypertrophy-focused training programs may be beneficial for elucidating potential hypertrophic differences between training conditions (e.g., LP versus UP, or periodized versus non-periodized RT). Similar to unloading phases, more studies should incorporate detraining cycles (i.e., periods of training cessation) into the periodized training plans, as research indicates that detraining phases may resensitize skeletal muscle to RT stimuli (Ogasawara et al., 2013). In this regard, it might be of interest to study the effects of various detraining/retraining cycles (e.g., 1 week of detraining followed by 3 weeks of retraining, or 2 weeks of detraining followed by 6 weeks of retraining) on muscle adaptations to periodized RT. The review by Afonso et al. (2017) found that many periodization studies do not control for nutritional intake. This is problematic, since differences in macronutrient intake (particularly protein) can affect the magnitude of muscle adaptations to RT (Morton et al., 2018). Finally, more research is required to determine the effects of various loading strategies (specifically, low-load training versus high-load training) on different muscle fibers, as this may have implications for periodized training designs aimed at maximizing skeletal muscle hypertrophy.

## Conclusion

The current body of evidence suggests a benefit of periodized RT programs for maximizing muscular strength in trained and untrained populations. Regarding the optimal periodization model for strength development, the research remains equivocal, although the undulating model may provide a superior effect. Furthermore, while the current data provide a basis for the use of periodized RT to optimize muscular strength, longer trials that incorporate taper periods and unloading phases are required to better assess the efficacy of periodized training. Regarding skeletal muscle hypertrophy, LP and UP appear to promote similar adaptations in untrained subjects; however, these findings are largely based on studies designed to maximize strength rather than hypertrophy. It is unclear whether periodized RT is able to enhance skeletal muscle hypertrophy beyond that of non-periodized programs.

## Author Contributions

The author confirms being the sole contributor of this work and has approved it for publication.

## Conflict of Interest Statement

The author declares that the research was conducted in the absence of any commercial or financial relationships that could be construed as a potential conflict of interest.
